# Supplementary material for: Spligation enables programmable chimeric RNA generation in living cells
Source: bioRxiv. 2026 Mar 6:2026.03.06.709984. Preprint. [Version 1] doi: 10.64898/2026.03.06.709984 (PMC13001472; doi:10.64898/2026.03.06.709984)
Supplement: Supplement 1 [file media-1.pdf]

Supplementary Information

Spligation enables programmable chimeric RNA generation in living cells

David Colognori*, Kevin Wasko*, Marena Trinidad*, Zehan Zhou, and Jennifer A. Doudna‡

*These authors contributed equally

‡Corresponding author: doudna@berkeley.edu

Supplementary Figures

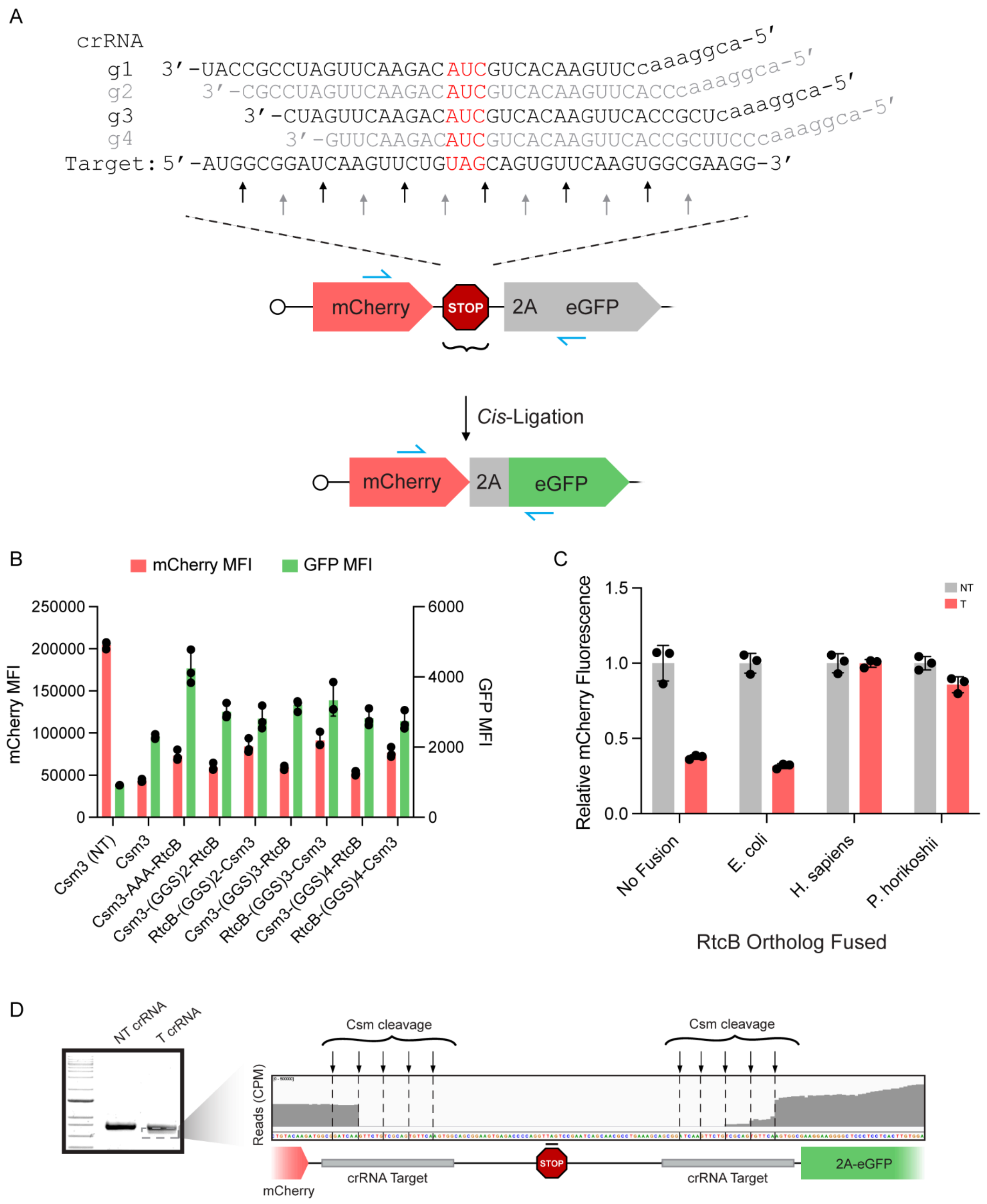

Supplementary Fig 1

A. Positioning of tiled crRNA sequences relative to the reporter construct stop codon region, corresponding to Figure 2C. Black arrows indicate possible cleavage sites for crRNAs 1 & 3, while gray arrows indicate possible cleavage sites for crRNAs 2 & 4.

B. Flow cytometry results from experiment testing fusion orientations & linker lengths. C-terminal Csm3 fusion with AAA linker performed the best and was used in all subsequent experiments.

C. Flow cytometry quantification of mCherry knockdown in RtcB fusion comparison experiment, corresponding to Figure 2E.

D. RNA was extracted from cells transfected with the construct shown in Fig. **2G** with the crRNA target sites spaced ~50 nt apart, reverse-transcribed into cDNA, and PCR amplified using the primers indicated in blue in (**Fig. 2G**). The PCR product was resolved on a 1.5% agarose gel and the indicated band excised and sequenced. Inset shows sequencing coverage across the Csm cleavage sites. Reporter sequence and diagram depicted below.

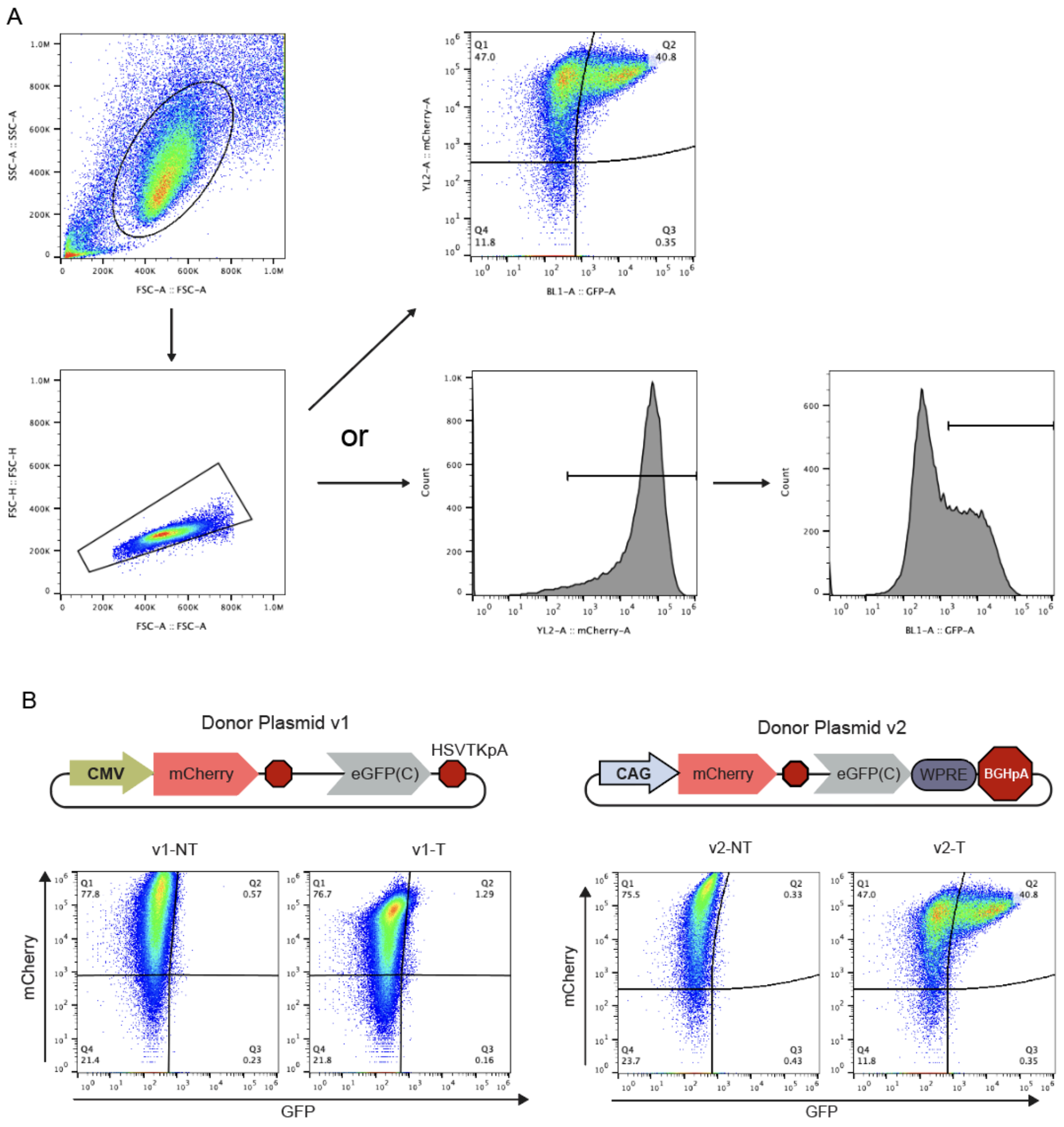

Supplementary Fig 2

A. Gating strategy for flow cytometry analysis, representative of all experiments.

B. Representative flow cytometry panels for trans-ligation experiments using original Donor-v1 (top) and enhanced Donor-v2 (bottom) constructs. Cells transfected with a non-targeting crRNA shown on left, targeting crRNA on right.

Ligation Product: XIST crRNA 2	N_Reads	Percent
CATGGCGGGCTGTGCTTTGTTAGGTTGTCCAA	550	75.86206897
CATGGCGGGCTGTGC------AGGTTGTCCAA	32	4.413793103
CATGGCGGG------TTTGTTAGGTTGTCCAA	30	4.137931034
CATGGCGGG------------AGGTTGTCCAA	21	2.896551724
CAT------------TTTGTTAGGTTGTCCAA	4	0.551724138
CATGGCGGG------------------TCCAA	2	0.275862069
CAT------------------AGGTTGTCCAA	1	0.137931034
CAT------CTGTGCTTTGTTAGGTTGTCCAA	0	0
CAT------------------------TCCAA	0	0
CATGGCGGGCTGTGC------------TCCAA	0	0
CATGGCGGGCTGTGCTTTGTT------TCCAA	0	0
Other_Reads	85	11.72413793
Total_Reads	725	100

Supplementary Table 1

Ligation Product: XIST crRNA 1	N_Reads	Percent
CGGATCCAGTTCTGTCGCAGTGTTCAAGTGGC	283	76.90217391
CGGATCCAG------------GTTCAAGTGGC	15	4.076086957
CGGATCCAGTTCTGT------GTTCAAGTGGC	9	2.445652174
CGGATCCAGTTCTGT------------GTGGC	14	3.804347826
CGGATCCAG------CGCAGTGTTCAAGTGGC	9	2.445652174
CGGATCCAG------------------GTGGC	7	1.902173913
CGG------TTCTGTCGCAGTGTTCAAGTGGC	0	0
CGG------------CGCAGTGTTCAAGTGGC	0	0
CGG------------------GTTCAAGTGGC	0	0
CGG------------------------GTGGC	0	0
CGGATCCAGTTCTGTCGCAGT------GTGGC	0	0
Other_Reads	31	8.423913043
Total_Reads	368	100

Supplementary Table 2

**Ligation Product: Cis 1-Cut Reporter**	**N_Reads**	**Percent**
GCGGATCAA------------------AGTGG	475	64.4504749
GCGGATCAAGTTCTGTAGCAGTGTTCAAGTGG	30	4.070556309
GCGGATCAA------TAGCAGTGTTCAAGTGG	56	7.598371777
GCGGATCAA------------TGTTCAAGTGG	24	3.256445047
GCGGATCAAGTTCTG------------AGTGG	15	2.035278155
GCG------------------------AGTGG	6	0.814111262
GCG------GTTCTGTAGCAGTGTTCAAGTGG	0	0
GCG------------TAGCAGTGTTCAAGTGG	0	0
GCG------------------TGTTCAAGTGG	0	0
GCGGATCAAGTTCTG------TGTTCAAGTGG	0	0
GCGGATCAAGTTCTGTAGCAG------AGTGG	0	0
Other_Reads	131	17.77476255
Total_Reads	737	100

Supplementary Table 3

Ligation Product: 2-Cut 100nt Reporter	N_Reads	Percent
GCGGATCAA-----------------------|---------------------------AGTGG	205	65.2866242
GCG-----------------------------|---------------------------AGTGG	1	0.318471338
GCGGATCAA-----------------------|---------------------TGTTCAAGTGG	11	3.503184713
GCGGATCAA-----------------------|---------------TCGCAGTGTTCAAGTGG	21	6.687898089
GCGGATCAAGTTCTGTCGCAG-----------|---------------------------AGTGG	1	0.318471338
GCGGATCAAGTTCTGTCGCAG-----------|---------------------TGTTCAAGTGG	5	1.592356688
GCG-----------------------------|---GATCAAGTTCTGTCGCAGTGTTCAAGTGG	0	0
GCG-----------------------------|---------GTTCTGTCGCAGTGTTCAAGTGG	0	0
GCG-----------------------------|---------------TCGCAGTGTTCAAGTGG	0	0
GCG-----------------------------|---------------------TGTTCAAGTGG	0	0
GCGGATCAA-----------------------|---GATCAAGTTCTGTCGCAGTGTTCAAGTGG	0	0
GCGGATCAA-----------------------|---------GTTCTGTCGCAGTGTTCAAGTGG	0	0
GCGGATCAAGTTCTG-----------------|---GATCAAGTTCTGTCGCAGTGTTCAAGTGG	0	0
GCGGATCAAGTTCTG-----------------|---------GTTCTGTCGCAGTGTTCAAGTGG	0	0
GCGGATCAAGTTCTG-----------------|---------------TCGCAGTGTTCAAGTGG	0	0
GCGGATCAAGTTCTG-----------------|----------------	0	0

-----TGTTCAAGTGG		
GCGGATCAAGTTCTG-----------------|----------------<br>-----------AGTGG	0	0
GCGGATCAAGTTCTGTCGCAG-----------|---GATCAAGTTCTGT<br>CGCAGTGTTCAAGTGG	0	0
GCGGATCAAGTTCTGTCGCAG-----------|---------GTTCTGT<br>CGCAGTGTTCAAGTGG	0	0
GCGGATCAAGTTCTGTCGCAG-----------|---------------T<br>CGCAGTGTTCAAGTGG	0	0
GCGGATCAAGTTCTGTCGCAGTGTTCA-----|---GATCAAGTTCTGT<br>CGCAGTGTTCAAGTGG	0	0
GCGGATCAAGTTCTGTCGCAGTGTTCA-----|---------GTTCTGT<br>CGCAGTGTTCAAGTGG	0	0
GCGGATCAAGTTCTGTCGCAGTGTTCA-----|---------------T<br>CGCAGTGTTCAAGTGG	0	0
GCGGATCAAGTTCTGTCGCAGTGTTCA-----|----------------<br>-----TGTTCAAGTGG	0	0
GCGGATCAAGTTCTGTCGCAGTGTTCA-----|----------------<br>-----------AGTGG	0	0
Other_Reads	70	22.29299363
Total_Reads	314	100

Supplementary Table 4

**Ligation Product: 2-Cut 300nt Reporter**	**N_Reads**	**Percent**
GCGGATCAA-----------------------|---------------------------AGTGG	148	48.05194805
GCGGATCAA-----------------------|---------------TCGCAGTGTTCAAGTGG	48	15.58441558
GCGGATCAA-----------------------|---------------------TGTTCAAGTGG	6	1.948051948
GCGGATCAAGTTCTGTCGCAG-----------|---------------------TGTTCAAGTGG	34	11.03896104
GCG-----------------------------|---------------------------AGTGG	1	0.324675325
GCG-----------------------------|---GATCAAGTTCTGTCGCAGTGTTCAAGTGG	0	0
GCG-----------------------------|---------GTTCTGTCGCAGTGTTCAAGTGG	0	0
GCG-----------------------------|---------------TCGCAGTGTTCAAGTGG	0	0
GCG-----------------------------|---------------------TGTTCAAGTGG	0	0
GCGGATCAA-----------------------|---GATCAAGTTCTGTCGCAGTGTTCAAGTGG	0	0
GCGGATCAA-----------------------|---------GTTCTGTCGCAGTGTTCAAGTGG	0	0
GCGGATCAAGTTCTG-----------------|---GATCAAGTTCTGTCGCAGTGTTCAAGTGG	0	0
GCGGATCAAGTTCTG-----------------|---------GTTCTGTCGCAGTGTTCAAGTGG	0	0
GCGGATCAAGTTCTG-----------------|---------------TCGCAGTGTTCAAGTGG	0	0
GCGGATCAAGTTCTG-----------------|---------------------TGTTCAAGTGG	0	0
GCGGATCAAGTTCTG-----------------|----------------	0	0

-----------AGTGG		
GCGGATCAAGTTCTGTCGCAG-----------|---GATCAAGTTCTGT CGCAGTGTTCAAGTGG	0	0
GCGGATCAAGTTCTGTCGCAG-----------|---------GTTCTGT CGCAGTGTTCAAGTGG	0	0
GCGGATCAAGTTCTGTCGCAG-----------|---------------T CGCAGTGTTCAAGTGG	0	0
GCGGATCAAGTTCTGTCGCAG-----------|---------------- -----------AGTGG	0	0
GCGGATCAAGTTCTGTCGCAGTGTTCA-----|---GATCAAGTTCTGT CGCAGTGTTCAAGTGG	0	0
GCGGATCAAGTTCTGTCGCAGTGTTCA-----|---------GTTCTGT CGCAGTGTTCAAGTGG	0	0
GCGGATCAAGTTCTGTCGCAGTGTTCA-----|---------------T CGCAGTGTTCAAGTGG	0	0
GCGGATCAAGTTCTGTCGCAGTGTTCA-----|---------------- -----TGTTCAAGTGG	0	0
GCGGATCAAGTTCTGTCGCAGTGTTCA-----|---------------- -----------AGTGG	0	0
Other_Reads	71	23.05194805
Total_Reads	308	100

Supplementary Table 5

**Ligation Product: Trans-Ligation Reporter**	**N_Reads**	**Percent**
GCGGATCAA-----------------------|---------------<br>TCGCAGTGTTCAAGTGG	1949	40.72294191
GCGGATCAA-----------------------|---------------<br>------TGTTCAAGTGG	364	7.605516089
GCGGATCAA-----------------------|---------------<br>------------AGTGG	1863	38.92603427
GCG-----------------------------|---------------<br>------------AGTGG	13	0.271625575
GCGGATCAAGTTCTG-----------------|---------------<br>------TGTTCAAGTGG	3	0.062682825
GCGGATCAAGTTCTG-----------------|---------GTTCTG<br>TCGCAGTGTTCAAGTGG	3	0.062682825
GCG-----------------------------|---------------<br>------TGTTCAAGTGG	1	0.020894275
GCG-----------------------------|---GATCAAGTTCTG<br>TCGCAGTGTTCAAGTGG	0	0
GCG-----------------------------|---------GTTCTG<br>TCGCAGTGTTCAAGTGG	0	0
GCG-----------------------------|---------------<br>TCGCAGTGTTCAAGTGG	0	0
GCGGATCAA-----------------------|---GATCAAGTTCTG<br>TCGCAGTGTTCAAGTGG	0	0
GCGGATCAA-----------------------|---------GTTCTG<br>TCGCAGTGTTCAAGTGG	0	0
GCGGATCAAGTTCTG-----------------|---GATCAAGTTCTG<br>TCGCAGTGTTCAAGTGG	0	0
GCGGATCAAGTTCTG-----------------|---------------<br>TCGCAGTGTTCAAGTGG	0	0
GCGGATCAAGTTCTG-----------------|---------------<br>------------AGTGG	0	0
GCGGATCAAGTTCTGTCGCAG-----------|---GATCAAGTTCTG	0	0

TCGCAGTGTTCAAGTGG		
GCGGATCAAGTTCTGTCGCAG-----------|---------GTTCTG TCGCAGTGTTCAAGTGG	0	0
GCGGATCAAGTTCTGTCGCAG-----------|--------------- TCGCAGTGTTCAAGTGG	0	0
GCGGATCAAGTTCTGTCGCAG-----------|--------------- ------TGTTCAAGTGG	0	0
GCGGATCAAGTTCTGTCGCAG-----------|--------------- ------------AGTGG	0	0
GCGGATCAAGTTCTGTCGCAGTGTTCA-----|---GATCAAGTTCTG TCGCAGTGTTCAAGTGG	0	0
GCGGATCAAGTTCTGTCGCAGTGTTCA-----|---------GTTCTG TCGCAGTGTTCAAGTGG	0	0
GCGGATCAAGTTCTGTCGCAGTGTTCA-----|--------------- TCGCAGTGTTCAAGTGG	0	0
GCGGATCAAGTTCTGTCGCAGTGTTCA-----|--------------- ------TGTTCAAGTGG	0	0
GCGGATCAAGTTCTGTCGCAGTGTTCA-----|--------------- ------------AGTGG	0	0
Other_Reads	590	12.32762223
Total_Reads	4786	100

Supplementary Table 6

Ligation Product: LBR	N_Reads	Percent
TACATCTAC------------------	3153	81.2628866
TACATCTACTAATGCTCTTCT------	549	14.14948454
TAC------TAATGCTCTTCTGGCTTT	0	0
TAC------------TCTTCTGGCTTT	0	0
TAC------------------GGCTTT	0	0
TAC------------------------	0	0
TACATCTAC------TCTTCTGGCTTT	0	0
TACATCTAC------------GGCTTT	0	0
TACATCTACTAATGC------GGCTTT	0	0
TACATCTACTAATGC------------	0	0
Other_Reads	178	4.587628866
Total_Reads	3880	100

Supplementary Table 7

Ligation Product: NCL	N_Reads	Percent
AAGTTTGAA------------------	2956	61.686
AAGTTTGAATAGCTT------------	967	20.179
AAG------------------------	73	1.5234
AAG------TAGCTTCTGTCCCTCTGC	0	0
AAG------------CTGTCCCTCTGC	0	0
AAG------------------CTCTGC	0	0
AAGTTTGAA------CTGTCCCTCTGC	0	0
AAGTTTGAA------------CTCTGC	0	0
AAGTTTGAATAGCTT------CTCTGC	0	0
AAGTTTGAATAGCTTCTGTCC------	0	0
Other_Reads	796	16.611
Total_Reads	4792	100

Supplementary Table 8

Ligation Product: NPM1	N_Reads	Percent
AAGTCTCTTTAAGAA------------	2082	43.17710494
AAGTCTCTT------------------	1827	37.8888428
AAG------TAAGAAAATAGTTTAAAC	0	0
AAG------------AATAGTTTAAAC	0	0
AAG------------------TTAAAC	0	0
AAG------------------------	0	0
AAGTCTCTT------AATAGTTTAAAC	0	0
AAGTCTCTT------------TTAAAC	0	0
AAGTCTCTTTAAGAA------TTAAAC	0	0
AAGTCTCTTTAAGAAAATAGT------	0	0
Other_Reads	913	18.93405226
Total_Reads	4822	100

Supplementary Table 9
